# A corroborative study on improving pitch determination by time–frequency cepstrum decomposition using wavelets

**DOI:** 10.1186/s40064-016-2162-0

**Published:** 2016-05-06

**Authors:** Fadoua Bahja, Joseph Di Martino, Elhassan Ibn Elhaj, Driss Aboutajdine

**Affiliations:** LRIT laboratory, Unit Associated to CNRST, URAC 29, Faculty of Sciences, Univrsité Mohammed V-Agdal, Avenue Ibn Batouta, B.P. 1014, Rabat, Morocco; LORIA, B.P. 239, 54506 Vandoeuvre-lès-Nancy, France; INPT, Avenue Allal Al Fassi, Madinat Al Irfane, Rabat, Morocco

**Keywords:** Wavelet transforms, Approximation coefficients, Cepstrum signal, Pitch estimation, Pitch tracking, Voicing decision

## Abstract

A new wavelet-based method is presented in this work for estimating and tracking the pitch period. The main idea of the proposed new approach consists in extracting the cepstrum excitation signal and applying on it a wavelet transform whose resulting approximation coefficients are smoothed, for a better pitch determination. Although the principle of the algorithms proposed has already been considered previously, the novelty of our methods relies in the use of powerful wavelet transforms well adapted to pitch determination. The wavelet transforms considered in this article are the discrete wavelet transform and the dual tree complex wavelet transform. This article, by all the provided experimental results, corroborates the idea of decomposing the cepstrum excitation by using wavelet transforms for improving pitch detection. Another interesting point of this article relies in using a simple but efficient voicing decision (which actually improves a similar voicing criterion we proposed in a preceding published study) which on one hand respects the real-time process with low latency and on the other hand allows obtaining low classifications errors. The accuracy of the proposed pitch tracking algorithms has been evaluated using the international Bagshaw and the Keele databases which include male and female speakers. Our various experimental results demonstrate that the proposed methods provide important performance improvements when compared with previously published pitch determination algorithms.

## Background

In speech processing, the cepstrum signal can be separated into the resonances of the vocal tract and the harmonic peaks coming from the source excitation. Pitch period estimation is an essential component in many speech processing applications: the production, analysis and perception of speech. This parameter is a principal indicator in phonetic, lexical, syntactic and emotional information. It involves the development of various techniques in advanced analysis and interpretation of speech signals. The pitch complexity appears in multitude pitch determination algorithms (PDAs) (Bagshaw et al. 1993; Bahja et al. 2012; Ben Messaoud et al. 2011; Kobayashu and Shimamura 1998; Noll 1967; Weiping et al. 2004; Hess 1983). These algorithms do not exhibit the same performance for all speech signals and in all conditions (Ben Messaoud et al. 2011; Hermes and Wiley 1993). Among pitch tracking complexities, we note: the variation of the fundamental frequency F0 in time (therefore pitch tracking in real-time is still difficult but desired); the appearance of harmonics that distorts the detection; the difficulty to realize the voiced/unvoiced decision on the pitch contours; and the difficulty to evaluate the pitch detector using an easy manageable database. The wavelet transforms provide a method for providing a solution to these problems and have been widely used in pitch tracking algorithms (Ben Messaoud et al. 2009; Nelson et al. 2008; Noll 1967; Weiping et al. 2004). The wavelet transform-based pitch period estimation assumes that the glottis closures are correlated with detect the maxima in the adjacent scales of the wavelet transform. For pitch period estimation, one needs to detect these maxima across these scales, which is often prone to errors especially in the case of noisy signals (Ghosh et al. 2007). Obaidat et al. (1998) applied the wavelet transform to estimate pitch period of synthetic signals and demonstrated that multiscale analysis based on a Gaussian window provides an alternative to classical wavelet detectors; moreover they suggest the use of subdyadic scales for analyzing signals corrupted by high levels of noise. In this paper, we present a new wavelet-based approach for estimating the pitch period. Our method has been evaluated using dedicated international databases and has been compared with different algorithms (Bahja et al. 2012; Kobayashu and Shimamura 1998; Noll 1967; Weiping et al. 2004). Hence we investigate in this article this new approach in order to estimate and track the pitch period of human speech signals. Kadambe and Faye Boudreaux-Bartels (1992) noted that the accuracy of the pitch period depends on the choice of the Wavelet Transform (WT). They developed a wavelet-based scheme for pitch detection and estimation and showed that the wavelet-based method is superior to traditional pitch estimation techniques. The problems of F0 detection concern firstly the change in time of F0, and secondly the appearance of harmonics that may distort detection. In this paper, we use wavelet transforms to estimate pitch period and we try to solve the problems mentioned above by applying the two discrete wavelet transforms (DWT and DT-CWT) to cepstrum excitation, in order to compute the elected pitch index. We used these two particular wavelet transforms because, on one hand, they are widely used at different scales for emphasizing different properties of the signal, and on the other hand, they have a great temporal resolution. The basic procedure of our new approach can be summarized by the following three steps:

- The extraction of the cepstrum excitation signal and the wavelet decomposition of this signal into 3 levels in order to obtain the approximation signals we enhance using a VisuShrink method (Donoho and Johnstone 1995) followed by a hard thresholding;
- An exhaustive search of the maximum peaks from the smoothed approximation signals in order to estimate the pitch period;
- Voiced/Unvoiced classification errors minimization using a simple voicing decision in order to track the pitch.

This article is organized as follows: “Background on WTs” section gives a general background concerning DWT, DT-CWT and cepstrum; “The ideas of the proposed approach in comparison with the Advanced Cepstrum (ACEP) method” section describes the main idea of our approach using the DWT and the DT-CWT decomposition of the cepstrum excitation signal; “The pitch period estimation” section details the pitch period estimation and the pitch tracking; “Voicing decisions and pitch tracking” section describes the decisions concerning voicing classification; “Experimental results” section gives experimental results using two international databases: the Bagshaw (Bagshaw et al. 1993) and the Keele databases (Plante et al. 1995), in order to evaluate the performance of proposed algorithms and finally “Conclusion” section concludes this work.

## Background on WTs

### DWT

**Fig. 1:**
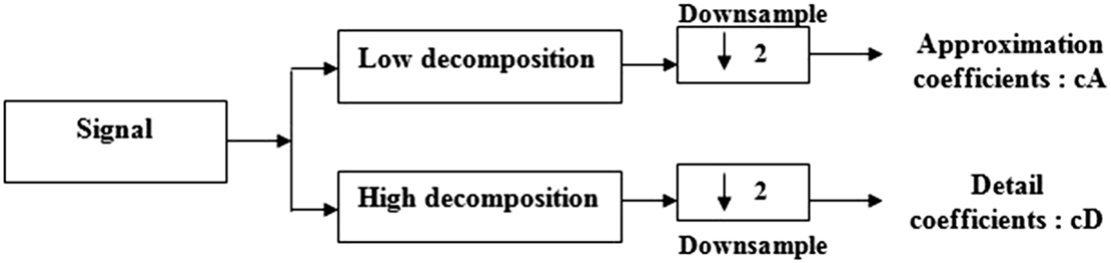
One-dimensional DWT

The DWT is computed by successive low pass and high pass filtering of the discrete time-domain signal as shown in Fig. 1. At each decomposition level, the high pass filter, followed by a down-sampling, produces detail coefficients *cD*, while the low pass filter, followed also by a down-sampling, produces approximation coefficients *cA* . At each decomposition level, the half-band filters produce signals covering only half the frequency band. The DWT properties are:

- Multi-resolution representation using a sub-band filter bank.
- The use of wavelets for iterating the filtering process at each level of decomposition.

### DT-CWT

**Fig. 2:**
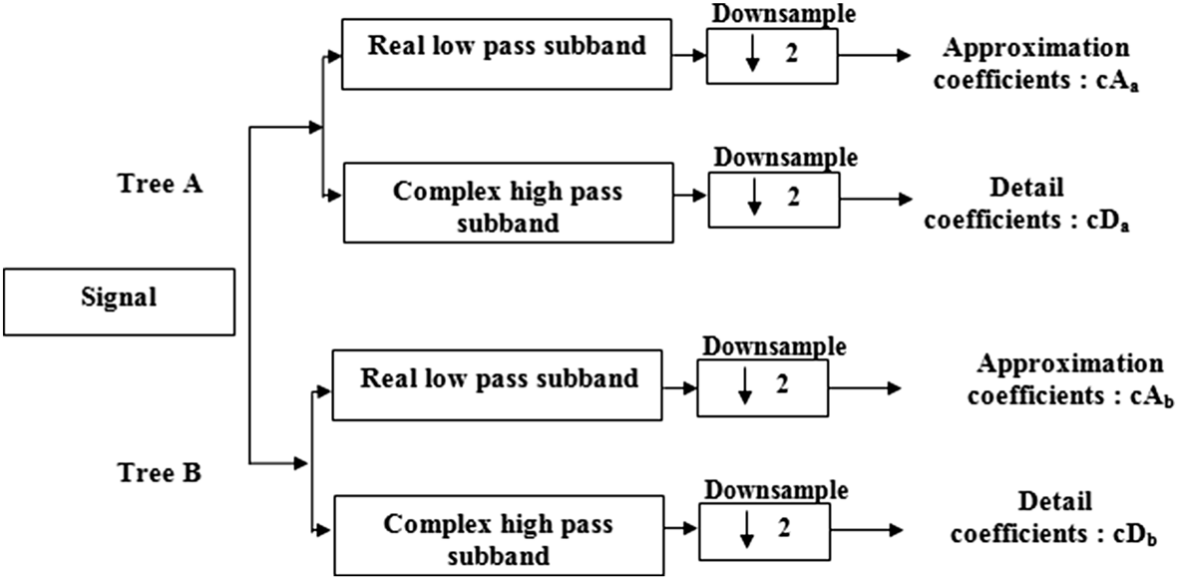
One-dimensional DT-CWT

The DT-CWT offers just another way to generate a time–frequency representation of the cepstrum excitation signal. The DT-CWT transform has been used successfully in many applications of signal and image processing (Kingsbury et al. 2004; Kwitt et al. 2010; Miller and Kingsbury 2008; Miller et al. 2005; Nelson et al. 2008). This transform is considered as an alternative of the classical DWT transform. Kingsbury introduced in Kingsbury (1998a, b) a complex wavelet transform, which allows an exact reconstruction of the analyzed signal. This transform has the property to be almost translation invariant. It should be noted that the invariance by translation can be obtained, with a bi-orthogonal transform, by doubling the sampling at each level of decomposition. Kingsbury proposed to obtain the quasi-translation invariant transform by doubling the sampling at the first level, then using filters and different sub-sampling in two different trees of decomposition. The coefficients at each scale are combined to form the complex coefficients. At level one, the two trees are shifted by one sample. The first one is formed by even coefficients and the other one by odd coefficients. Each tree is decomposed into a real low pass filter of odd length and a complex high pass filter of odd length. For the other levels, the two trees are shifted by half a sample. This is made possible by using different decompositions by DT-CWT. The complex transform of a signal is provided using two separate DWT decompositions (tree A and tree B). Figure 2 shows one level of wavelet decomposition by DT-CWT. The complex transform of a signal is provided using two separate DWT decompositions (tree A and tree B). The role of these trees is to produce respectively the real and imaginary coefficients.

The DT-CWT properties are:

- The shift is nearly invariant;
- The decomposition is directionally selective in two and higher dimensions;
- The multidimensional is non separable.

### The cepstrum signal

The cepstrum signal is composed of two parts:

- The first one concerns the excitation cepstrum located in the high quefrencies.
- The second one concerns the vocal tract cepstrum located in the low quefrencies.

The cepstrum signal is obtained by the following steps:

1. An Hamming window is applied to the short-time input signal in order to reduce the discontinuities at the boundaries;
2. An FFT (Fast Fourier Transform) and a modulus operators are applied then to this windowed signal in order to obtain an amplitude spectrum;
3. A log operator is then applied;
4. Finally an IFFT (Inverse Fast Fourier Transform) is applied to this log amplitude spectrum in order to obtain the cepstrum signal.

**Fig. 3:**
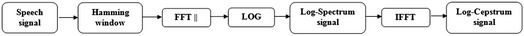
Flowchart of the log-cepstrum computation

As explained below below, Fig. 3 provides a block diagram showing how the log-cepstrum signal is computed.

## The ideas of the proposed approach in comparison with the Advanced Cepstrum (ACEP) method

**Fig. 4:**
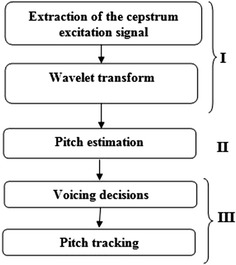
The proposed approach

The four major differences between our approach and the ACEP method (Weiping et al. 2004) which uses a similar decomposition technique using wavelets are: (1) The used wavelet transforms by the ACEP method is the dyadic wavelet transform. This wavelet transform does not use the downsampling operator such as DWT or DT-CWT, which implies an unclear appearance of the peaks. (2) The ACEP method applies the wavelet transform on the log excitation spectrum, contrary to our approach which applies directly the wavelet transform on the cepstrum excitation where peaks appear clearly. (3) The ACEP method uses only one decomposition level (the third one) for maximum peak searching, contrary to our method which uses (in order to obtain a robust pitch extraction) all the decomposition levels for maximum peak searching. (4) More importantly pitch tracking has not been considered by the ACEP method. The main ideas of our approach are summarized in Fig. 4.

The block diagram shown in Fig. 4 details the three main steps of our algorithm:

- Step 1: From the cepstrum signal, we can easily separate the vocal tract signal (which is located in the low quefrencies), from the excitation cepstrum located in the high quefrencies. Then we apply to the excitation part of the cepstrum signal a wavelet transform in order to obtain the approximation coefficients we enhance.
- Step 2: From the enhanced approximations, we estimate the pitch period;
- Step 3: Simple but efficient voiced/unvoiced decisions are carried out in order to track the pitch period.

### Extraction of the cepstrum excitation

**Fig. 5:**
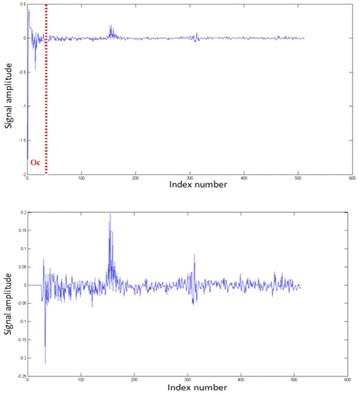
*Above* The log-cepstrum signal; *below* the log-cepstrum excitation signal

The cepstrum signal can be used to separate the excitation signal (which contains the pitch) from the transfer function (which contains the vocal tract information). From the log-cepstrum, we set to zero the first coefficients representing the vocal tract information. In our experiments, Oc is the number of vocal tract cepstral coefficients. Figure 5 describes the cepstrum excitation signal by zeroing the *Oc* first vocal tract coefficients. In our study we fix *Oc* to 20. That means that for a sampling frequency of 20 kHz, the maximum pitch frequency which can be determined is 1000 Hz (20 kHz/Oc). The cepstrum excitation signal is useful in speech processing domain and especially in pitch determination algorithms, because the low-frequency periodic excitation can be extracted from it. Human pitch detectors assume that pitch frequency is between 50 and 600 Hz generally. Trying to detect higher pitch frequency may introduce errors in the extraction. The fact that our algorithms can determine (theoretically) pitch frequency up to 1000 Hz, with low classification errors, is another proof of the high precision of our pitch detectors.

### Enhancement of the approximation coefficients

**Fig. 6:**
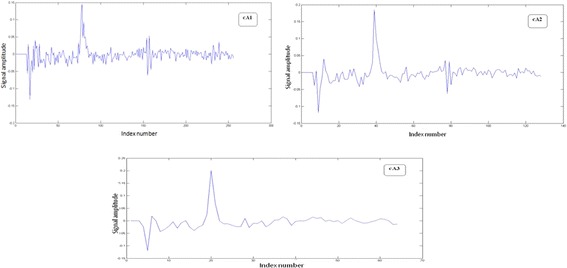
Approximation coefficients for level 1, 2 and 3 respectively without thresholding

The wavelet transform is applied to the log-cepstrum excitation signal in order to minimize the classification errors using a number of levels, which we experimentally fixed to 3 (see Fig. 6). In the 3 decomposition levels, we get 3 maximum peaks: we choose the maximum of these 3 maxima values as the elected maximum peak.

**Fig. 7:**
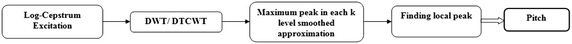
Flowchart of the DWT and DT-CWT decomposition for the cepstrum excitation signal

The main idea of our approach consists in smoothing these coefficients, in order to better estimate the pitch period given by the local maximum peak index at each decomposition level (see Fig.  7).

**Fig. 8:**
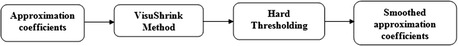
Smoothing the approximation coefficients

After applying a DWT using Haar filters or a DT-CWT transform, a hard thresholding is used to enhance the approximation coefficients: the coefficients below a threshold T1 are replaced by zero and in consequence the approximation coefficients are smoothed (Fig. 8). Approximations are consequently improved and provide an enhanced frequency resolution.

The literature proposes several methods for signal denoising such as VisuShrink (Donoho and Johnstone 1995), SureShrink (Donoho et al. 1995) and BayesShrink (Chang et al. 2000). In our approach we use the VisuShrink method for its simplicity and its effectiveness. The goal of using VisuShrink thresholding is to minimize the probability that any noise sample will exceed a certain threshold. This threshold is given by Formula 1 and 2:

1 $$\begin{aligned} \sigma = \frac{median(|cA|)}{0.6745} \end{aligned}$$

where:

- *cA* represents the approximation coefficients for the DWT and DT-CWT decompositions. The *cA* coefficients allow to obtain the maximum peak index in the real low pass sub-band;
- The factor 0.6745 in the denominator rescales the numerator in order to make $\sigma$ a suitable estimator for the standard deviation.

Donoho et al. (1995) proposed the universal threshold to use in VisuShrink method (Donoho and Johnstone 1995):

2 $$\begin{aligned} T1=\sigma \sqrt{2\log N } \end{aligned}$$

where *N* is the length of the analysis window at each level. Hard thresholding sets any coefficient less than or equal to the threshold *T*1 to zero as specified by Formula 3:

3 $$\begin{aligned} {\mathbf{if }}\, |cA[i][k]| <= T1 \Rightarrow cA[i][k] = 0.0 \end{aligned}$$

where *i* represents the decomposition level (1, 2 or 3); *k* is an index of a particular coefficient of *cA*[*i*] ; and *T*1 , is the universal threshold given by Formula 2. The hard thresholding technique reduces the estimation error in each coefficient. In this way, the aperiodic components in the input excitation for example due to aspiration or ambient noise are removed while at the same time preserving the slow and rapid variations in the underlying waveform. This is possible because of the compactness property of wavelets (i.e. localization in time). Our new approach consists in searching the pitch period in the wavelet-smoothed approximation coefficients. The calculation of these coefficients by the DWT with Haar filters or the DT-CWT provides a more robust peak index determination related to the pitch period.

## The pitch period estimation

To estimate the pitch period, a search is performed in order to find the highest value in the smoothed wavelet coefficients, which gives the peak related to the pitch frequency. According to the following Formula 4, the maximum peak index is related to the pitch by:

4 $$\begin{aligned} Pitch(j)=\frac{F_{s}}{I_{max}(j)}Hz \end{aligned}$$

where:

- *Fs* is the sampling frequency;
- $I_{max}(j)$ is the maximum peak index given by the highest signal amplitude of the 3 decompositions levels related to the *j*th analyzed frame.

## Voicing decisions and pitch tracking

In this section, we present a smart and easy technique for voicing decision, which respects real-time and uses only the preceding frames in order to track the pitch. In a speech signal, most of the voiced regions contain speech or speaker specific attributes, while silences or background noises are completely undesirable. However, we have to know if the cepstrum excitation signal exhibits periodic peaks (voiced regions) or random ones (unvoiced regions). The role of the pitch tracking algorithm is to detect correctly the voiced/unvoiced speech components.

### The voicing decision

In this section, we present the voicing decision method used for estimating the pitch. We can solve this issue by using a threshold in order to decide if the current frame is voiced or unvoiced (Rabiner and Sambur 1977). We apply Formula 5 on the maximum peak indexes whose determination has been explained previously. Such an Euclidian distance ignores the correlations between each pitch period and treats each maximum peak index equally. When the region is voiced, the indexes of the maximum peaks vary slowly. Formula 5 gives the first thresholding concerning the quantity *S* using an experimentally-determined threshold *T*2 .

5 $$\begin{aligned} S(j)=\sqrt{\sum _{k=0}^{L-1} [I_{max}(j-k) - I_{max}(j-k-1)]^{2}} \end{aligned}$$

where *L* is the number of preceding considered frames (for our experiment $L = 10$). The second criterion concerns the calculation of the signal energy of the windowed frame, which is considered as a sufficient condition to better decide if the frame is voiced or unvoiced. The signal energy *E*(*j*) of a particular frame *j* is defined as the log sum of the squared values of the signal samples. For a particular frame whose speech signal (windowed by a Hamming window) is below an experimentally determined threshold *T*3, we classify it as an unvoiced frame, otherwise it is considered as possibly voiced. Consequently our voiced/unvoiced decision is given by Formula 6:

6 $$\left\{ {\begin{array}{*{20}l} {{\mathbf{if}}\;E(j) < T3,} \hfill & {{\text{the}}\;jth\;{\text{frame}}\;{\text{is}}\;{\text{unvoiced}}} \hfill \\ \,{{\mathbf{else}}\;{\mathbf{if}}\;S(j) < T2,} \hfill & {{\text{the}}\;jth\;{\text{frame}}\;{\text{is}}\;{\text{voiced}}} \hfill \\ \, {{\mathbf{else}}} \hfill & {{\text{the}}\;jth\;{\text{frame}}\;{\text{is}}\;{\text{unvoiced}}} \hfill \\ \end{array} } \right\}$$

where *T*2 in our experiments is 10 and *T*3 is 76 dB. These thresholds have been determined experimentally.

### Corrections

In pitch tracking, the results obtained can detect false estimations of the F0 by the presence of parasitic peaks or valleys. When thresholding criteria failed, we correct the pitch contour from parasitic peaks and valleys by using the following technique:

- Starting with isolated peaks: the pitch peak is eliminated if its duration is bellow 13.5 ms (which corresponds to 9 frames). The shift used between two consecutive frames, in our study, is 1.5 ms (30 samples).
- Concerning valleys: we rebuilt the pitch contour linearly if the duration of the valley is also below 13.5 ms. We propose to regularize the pitch tracking in respecting the real time process. So emphasis is placed on the very low latency obtained which is 13.5  ms.

## Experimental results

The test of the proposed approach and the voicing decision used were evaluated over the Bagshaw (Bagshaw et al. 1993) and Keele (Plante et al. 1995) databases.

### The databases

*The Bagshaw database* Paul Bagshaw’s database was recorded at the University of Edinburgh (Centre for Speech Technology Research) and authored by Paul Bagshaw. The speech and laryngograph signals of this database were sampled at 20 kHz. It contains 0.12 h of speech, 50 English sentences each pronounced by one male and one female speaker. The fundamental frequency was computed by estimating the location of the glottal pulses in the laryngograph data and taking the inverse of the distance between each pair of consecutive pulses. Each fundamental frequency estimate is associated to the time instant in the middle of the pair of pulses used to derive the estimate.

*The Keele database* The Keele Pitch Database was recorded at Keele University. Data were collected for five male and five female English speakers, each of them read a phonetically balanced text: the “north-wind story”. The speech and laryngograph signals were sampled at 20 kHz. The fundamental frequency was estimated by applying an autocorrelation on windows of 25.6 ms shifted by intervals of 10 ms.

### Tracking the pitch period

**Fig. 9:**
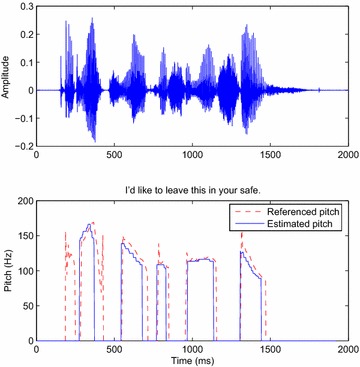
*Above* Input signal; *below* the pitch tracked by the DWT decomposition algorithm (*blue curve*) and the reference contour extracted from the database for a male speaker (*red curve*)

**Fig. 10:**
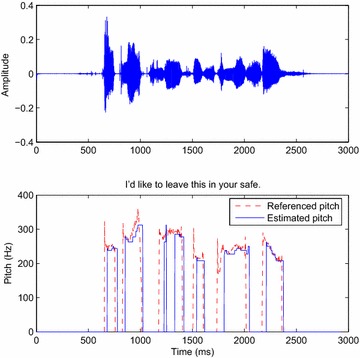
*Above* Input signal; *below* the pitch tracked by the DWT decomposition algorithm from a female speaker

Figures 9 and 10 (respectively 11 and 12) exhibit the pitch tracked by the DWT (DT-CWT) decomposition algorithm respectively for a male and a female utterance of the Bagshaw database using the voicing decision method detailed in the previous section. The plotting of the estimated pitch period extracted by the DWT approach, after corrections, indicates some remaining tracking errors.

**Fig. 11:**
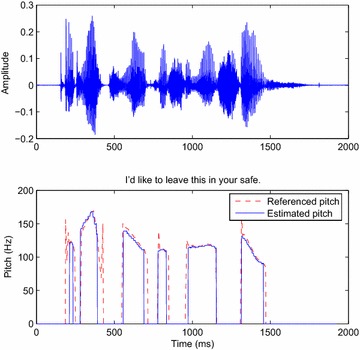
*Above* Input signal; *below* the pitch tracked by the DT-CWT decomposition algorithm from a male speaker

**Fig. 12:**
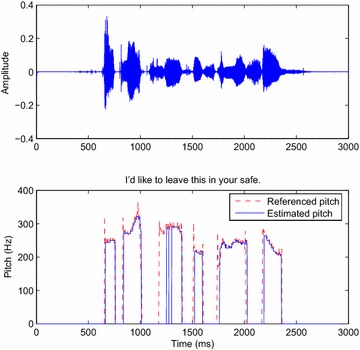
*Above* Input signal; *below* the pitch tracked by the DT-CWT decomposition algorithm for a female speaker

### Results

The length of the analyzed frame used is 51.2 ms and the number of decomposition levels is 3. The proposed approach is effective if the evaluation of the pitch period is related to a reliable voicing decision. Thus, the assessment of gross error rate (GER) implies the evaluation of the classification error (CE) (see Formula 7). For estimating the pitch detector performance, we compare the pitch reference given by the electroglottographic signal with the pitch contours provided by our algorithm. The evaluation criteria are measured according to the following classification errors (Chu and Alwan 2009, 2012):

- Classification Error (CE) is the percentage of unvoiced frames classified as voiced plus the percentage of voiced frames classified as unvoiced (Chu and Alwan 2009).

7 $$\begin{aligned} CE=\frac{N_{UV\rightarrow V}+ N_{V\rightarrow UV}}{\text{ N }} \end{aligned}$$

where:

- $N_{UV\rightarrow V}$ is the number of unvoiced frames classified as voiced;
- $N_{V\rightarrow UV}$ is the number of voiced frames classified as unvoiced;
- N is the total number of frames in the utterances.

- Gross Error Rate (GER): percentage of voiced frames with an estimated F0 value that deviates from the reference value more than 20 %. When the error is less than −20 %, it is counted as a gross error low; errors exceeding +20 % are counted as gross error high.
- Mean is the mean of the absolute differences between the reference and the estimated fundamental frequency values.
- Standard Deviation (SD) is the standard deviation of the absolute differences between the estimated and reference pitch values.

Tables 1 and 2 summarize the evaluation of DWT and DT-CWT, in comparison with recent related algorithms using the Bagshaw database. The algorithms tested are summarized below:

- CEP is the cepstrum-based pitch reference estimation algorithm (Noll 1967) for extracting the pitch as the frequency whose inverse maximizes the cepstrum signal. CEP is concerned by the problem of harmonics and also by the maximum F0 value it can detect.
- MCEP is the Modified CEP (Kobayashu and Shimamura 1998) which introduces the “clipping” method for removing the high frequencies in order to provide a solution of the noise problem. By using an IFFT the pitch period is extracted from the cepstrum signal.
- ACEP is the Advanced CEP (Weiping et al. 2004), which carries out a 3 levels wavelet transform.
- WCEPD for Wavelet and Cepstrum Excitation for Pitch Determination (Bahja et al. 2012) is a pitch tracking method based on a wavelet transform in the temporal domain. It is designed to estimate the pitch period of the speech signal from the cepstrum excitation signal processed by a wavelet transform.
- eCATE++ for enhanced Circular Autocorrelation of the Temporal Excitation (Bahja et al. 2013) is an algorithm for pitch detection based on an implicit circular autocorrelation of the glottal excitation signal.
- DWT and DT-CWT concern the two wavelet algorithms used in our approach under the voicing decision presented above.

**Table 1:** CE, GER and Abs-deviation for the male corpus of the Bagshaw database

Method	CE %	Gross error		Abs-deviation	
		Low (%)	High (%)	Mean (Hz)	SD (Hz)
CEP	0.27	1.11	2.96	3.51	3.76
MCEP	0.23	0.65	0.88	2.41	2.98
ACEP	0.14	1.16	0.25	2.31	3.01
WCEPD	0.11	0.41	0.06	3.15	2.84
eCATE++	*0.08*	0.27	0.71	*1.82*	2.91
DWT	0.13	0.31	0.01	3.01	2.56
DT-CWT	0.16	*0.24*	*0.00*	2.06	*2.29*

**Table 2:** CE, GER and Abs-deviation for the female corpus of the Bagshaw database

Method	CE (%)	Gross error		Abs-deviation	
		Low (%)	High (%)	Mean (Hz)	SD (Hz)
CEP	0.23	1.46	3.07	10.68	9.39
MCEP	0.17	0.99	1.94	8.45	7.89
ACEP	0.10	1.04	0.54	8.38	7.63
WCEPD	0.17	0.54	0.22	10.86	7.29
eCATE++	*0.06*	*0.31*	0.39	*4.27*	5.50
DWT	0.15	0.38	0.31	10.37	6.37
DT-CWT	0.14	0.39	*0.22*	6.48	*5.42*

The best results in each column for each table are indicated in italic. The proposed algorithm based on DT-CWT exhibits the lowest gross error high rates and the lowest absolute standard deviations for the male and female corpora of the Bagshaw database. But an important improvement achieved by our new approach based on DT-CWT concerns the 0 % gross error high rate for the male corpus. The presented algorithm compares favorably with other established methods and could be useful in real-time applications where a very low latency and a good pitch detection accuracy are absolutely necessary. For the Keele database, Tables 3 and 4 summarize the evaluation of DWT and DT-CWT, in comparison with: SWIPE (Chu and Alwan 2009), SPM (Ben Messaoud et al. 2011), CSAPM (Ben Messaoud et al. 2012), YIN (De Cheveigné and Kawahara 2002), CEP (Noll 1967), PRAAT (Krusback and Niederjohn 1991) and eCATE++ (Bahja et al. 2013) pitch algorithms. In order to compare the performance of pitch determination algorithms, we propose to estimate the voicing decision results using the following error rates:

- The Gross Pitch Error (GPE) (Nakatani et al. 2008):
  8 $$\begin{aligned} GPE=\frac{N_{GE}}{N_{vv}}*100\,\% \end{aligned}$$
  where $N_{vv}$ is the number of frames considered as voiced both from the pitch tracker and the reference pitch contours; *vv* means both voiced; and
  $N_{GE}$ is the number of voiced frames for which $|\frac{F0_{i,estimated}}{F0_{i,reference }}-1| > 0.2$ where *i* is the frame number.

We also calculate the following rate:

- F0 Frame Error (FFE) metric (Nakatani et al. 2008):
  9 $$\begin{aligned} FFE=\frac{N_{vv}}{N}*GPE+CE \end{aligned}$$
  It sums the three types of errors mentioned above:
  10 $$\begin{aligned} FFE=\frac{N_{V\rightarrow UV}+ N_{UV\rightarrow V}+N_{GE}}{N}*100\,\% \end{aligned}$$

**Table 3:** GPE rates for pitch estimation using Keele University database

PDA	GPE (%)		
	Male speakers	Female speakers	Mean
CEP	3.7	4.2	3.95
PRAAT	2.9	3.3	3.1
YIN	3.5	1.2	2.35
eCATE++	0.48	0.40	0.44
DWT	0.38	0.34	0.36
DT-CWT	*0.37*	*0.30*	*0.33*

According to the experimental results exhibited in Tables 3 and 4, we can say that our approach reaches very good results either for the male or female corpora of the Keele database. The lowest classification error rates obtained using the Bagshaw or Keele databases clearly demonstrate the effectiveness of the proposed approach.

**Table 4:** Performance of PDAs using the Keele database

PDA	GPE (%)	CE (%)	FFE (%)
YIN	2.28	6.28	7.23
SWIPE	0.62	3.92	4.19
SPM	0.75	3.02	3.31
CSAPM	0.67	2.27	2.59
eCATE++	0.44	*0.65*	1.55
DWT	0.36	0.78	1.41
DT-CWT	*0.33*	0.81	*1.39*

The DWT decomposition does not have a translation invariant property. Therefore, results related to this algorithm may be affected when compared to those obtained by a translation invariant method. To overcome this drawback, we use the DT-CWT decomposition, which is translation invariant. The experimental results obtained exhibit the influence of this property. In order to summarize the results, Table 5 shows clearly that the DWT and the DT-CWT algorithms outperforms numerous important reference pitch determination algorithms tested on the two databases (Baghshaw and Keele). To make this comparison possible, we calculated the MFPE (Mean of Fine Pitch Error) which measures the bias of the *F*0 estimation when no gross estimation error occurred (Bahja et al. 2013; Chu and Alwan 2012).

11 $$\begin{aligned} MFPE=\frac{1}{N_{FE}}\sum \limits _{{i\in S_{FE}}}^{}(F0_{i,estimated} - F0_{i,reference }) \end{aligned}$$

where $S_{FE}$ denotes the set of all the frames in which no gross error occurs and $N_{FE}$ (for “Fine Error”) is equal to $N_{vv} - N_{GE}$.

**Table 5:** GPE and MFPE for algorithms using the Keele and the Bagshaw corpora

	Keele database		Bagshaw database	
PDA	GPE (%)	MFPE (Hz)	GPE (%)	MFPE (Hz)
CPD	3.95	–	4.65	–
eSRPD	3.90	–	1.40	–
PRAAT	3.10	0.19	2.27	−0.77
YIN	2.35	0.55	2.25	−0.39
RAPT	2.62	0.79	2.45	−0.06
SAFE	2.98	−0.36	2.45	−1.39
eCATE++	0.44	−*0.03*	0.81	−1.67
DWT	0.36	−0.26	*0.25*	−2.39
DT-CWT	*0.33*	−0.11	*0.25*	−*0.52*

## Conclusion

The presented work focuses especially on the estimation of the pitch period, the pitch tracking algorithm and the voiced/unvoiced decision in real-time. This study corroborates the idea of decomposing the cepstrum excitation signal using powerful wavelet transforms such as DWT or DT-CWT for improving pitch determination. The main contributions of the presented algorithm consists in obtaining a very low latency (13.5 ms), which must be compared with the latency obtained by the eCATE++ algorithm (20.25 ms), and low classification errors for both the Bagshaw and Keele databases.
